# ‘Modelling social exclusion in a diagnostically-mixed sample of people with severe mental illness’

**DOI:** 10.1177/00207640211001893

**Published:** 2021-03-17

**Authors:** Gillian Mezey, Sarah White, Isobel Harrison, Jennifer Bousfield, Helen Killaspy, Brynmor Lloyd-Evans, Sarah Payne

**Affiliations:** 1 St George’s University of London, UK; 2 University College London, UK; 3 University of Bristol School of Applied Community and Health Studies, UK

**Keywords:** Social inclusion, mental illness, psychosis, common mental disorder, personality disorder

## Abstract

**Background:**

Social inclusion is an important indicator of recovery in individuals with severe mental illness. The Social Inclusion Questionnaire User Experience (SInQUE) is a new measure of social inclusion for mental health service users which assesses five domains (consumption, production, access to services, social integration and civil engagement). It has good psychometric properties and is acceptable to service users and mental health professionals. It is not clear whether individuals with different diagnostic conditions experience a similar reduction in social inclusion.

**Aims:**

(1) Investigate whether current social inclusion differs between diagnostic groups (people with schizophrenia/other psychotic disorders, common mental disorder or personality disorder); (2) Identify factors associated with lower social inclusion; (3) Examine associations between social inclusion and stigma, quality of life and loneliness.

**Method:**

Mental health service users with psychotic disorder, personality disorder or common mental disorder, living in the community, completed the SInQUE, alongside other validated outcome measures. Multiple regression investigated associations.

**Results:**

About 192 service users (55% with psychotic disorder; 26% with common mental disorder; 19% with personality disorder). Current social inclusion did not vary according to diagnosis, except for the sub-domain of productivity, where individuals with personality disorder were more socially included than the other two groups. Lower social inclusion was associated with older age (*p* = .008), lack of higher education (*p* < .001), more previous admissions (*p* = .005), severity of current symptoms and greater experienced stigma (*p* = .006) and anticipated stigma (*p* = .035). Greater social inclusion was associated with better quality of life (*p* < .001) and less loneliness (*p* < .001).

**Conclusions:**

Barriers to social inclusion in individuals with severe mental health problems include factors related to the illness, such as symptom severity and external factors, such as stigma and discrimination. Social inclusion is a recovery goal and should be routinely assessed. Increasing people’s social inclusion benefits service users in terms of improved mental health, better quality of life and reduced loneliness.

## Background

Social inclusion refers to the ability of an individual to participate in the key activities of society, that they would like to participate in, for reasons that are outside their control (Burchardt et al., 2002; Morgan et al., 2007).

People with mental health problems have higher rates of educational drop-out and disruption (Isohanni et al., 2001), unemployment (Boardman et al., 2003; Harvey et al., 2009; Marwaha et al., 2007; Meltzer at al., 2002), poverty and debt compared to the general population. They are more likely to experience negative life events, including criminal victimisation (Johnson et al., 2015; Khalifeh & Dean, 2010; Teplin et al., 2005; Walsh et al., 2003), they tend to lack social support (Meltzer et al., 2002) and have fewer and poorer quality social networks compared to the mentally well (Bengtsson-Tops & Hansson, 2001; Sweet et al., 2018). They are less likely to be in a stable relationship (Thomas & Randall, 2012) and commonly report feelings of loneliness and isolation (Alasmawi et al., 2020; Meltzer et al., 2013; Wang et al., 2018).

Such experiences represent aspects of, and are risk factors for, lower levels of social inclusion.

Increased social inclusion has been found to be associated with improved mental health and wellbeing (Boardman, 2011; Morgan et al., 2007; Office of the Deputy Prime Minister, 2004; Rankin, 2005; Sweet et al., 2018). Low levels of social inclusion, sometimes referred to as social exclusion, is a risk factor for poor mental health and the development of, or exacerbation of pre-existing psychiatric disorder (Boardman, 2011; Morgan et al., 2007; Office of the Deputy Prime Minister, 2004).

The association between social exclusion and poor mental health may be as a result of factors directly related to the illness, such as symptoms of paranoia, anxiety, low mood, amotivation, cognitive impairments, interpersonal difficulties and side effects of medication (Burns & Patrick, 2007) as well as to external factors such as poverty, stigma and discrimination (Mezey et al., 2016; Thornicroft et al., 2007).

The Social Inclusion Questionnaire User Experience (SInQUE) was developed by the authors to assess social inclusion across five domains; consumption, production, access to services, social integration and civil engagement (Mezey et al., 2020, 2013). The higher the score, the greater the level of social inclusion. The tool is completed by a member of staff through discussion with the service user. It has been shown to have good psychometric properties and to be acceptable to both service users and mental health professionals (Mezey et al., 2020, 2013). Whilst previous studies have assessed the social inclusion of people with severe mental illness, none have assessed people with other types of mental health problem using a standardised measure.

We have previously reported that individuals with severe psychotic illness, tend to experience worsening social inclusion over time (Killaspy et al., 2014; Mezey et al., 2013). This study aimed to investigate whether social inclusion differs between diagnostic groups (people with a primary diagnosis of schizophrenia/other psychotic disorders, common mental disorder or personality disorder). We also aimed to identify factors associated with lower levels of social inclusion and to examine associations between social inclusion and stigma, quality of life and loneliness, as a way of developing more targeted support and interventions to improve social inclusion.

We tested four hypotheses. First, that levels of social inclusion would differ between diagnostic groups. Second, that lower levels of social inclusion would be associated with higher levels of stigma and discrimination (Rüsch et al., 2011). Third, that individuals with the most severe symptoms would encounter the most stigma and discrimination, resulting in lower social inclusion, compared to individuals with less severe symptoms. Finally, that greater social inclusion would be associated with better quality of life (Mezey et al., 2020) and less loneliness across all three diagnostic groups.

## Methods

### Procedures

The study was approved by the London–Bromley Research and Ethics Committee (ref IS/LO/1778).

This was a mixed methods cross-sectional study, including the collection of retrospective data. Quantitative data were collected through individual structured interviews with service users and case records of service users were reviewed to obtain or confirm socio-demographic data and psychiatric contacts.

An information sheet about the study was sent to the managers and consultant psychiatrists of the community mental health services in the two Trusts. Team members were asked to identify service users who met the inclusion criteria and could be approached to take part in the study. If the service user agreed to be contacted about the study, their name was passed to one of the researchers (JB, IH), who then arranged to meet them, usually at the community team base or at their home. The service user was given a participant information sheet about the study and had an opportunity to ask questions about the study before providing written informed consent.

All service users received £20 for each research interview in recognition of their time.

### Participants and setting

Recruitment took place between December 2015 and May 2017.

#### Service users

Service user participants were recruited from community mental health services in South West London and St Georges NHS Mental Health Trust (SWLSTG) and Camden and Islington NHS Foundation Trust (C & I). Participating teams included community mental health teams, community rehabilitation teams, complex depression and trauma teams, personality disorder services, assertive outreach teams, early intervention for psychosis services and community forensic services.

All participants had to be over 18 years old and be able to speak and understand English. Additional inclusion criteria were: a primary diagnosis of a psychotic illness (e.g. schizophrenia, schizoaffective disorder or bipolar affective disorder), common mental disorder (depression, anxiety, obsessive compulsive disorder, posttraumatic stress disorder) or personality disorder; currently receiving treatment from one of the community teams listed above; at least one previous inpatient admission or a period of care from a Crisis Resolution/Home Treatment Team and a period of at least 3 months living in the community, since last inpatient admission.

### Measures

Data on service users were collected through a face-to-face structured interview with a researcher, using validated self-report scales and case note review.

Sociodemographic details and information about the person’s psychiatric history included: age; gender; ethnicity; current civil status; educational attainment; current employment status; diagnosis (ICD-10); duration of contact with psychiatric services. Case notes were reviewed to gain details of contacts with staff in the last year.

#### SInQUE

The SInQUE is completed through a structured interview and comprises items that provide scores on five domains: productivity (PRO); consumption (CON); access to services (SA); social integration (SI); and political engagement (POL) (Mezey et al., 2013). Ratings can be recorded in relation to the year prior to the service user’s first admission to hospital, or treatment from a Home Treatment/crisis team (T1-32 items) and in the past year (T2-58 items). Only the T2 section of the tool was used in this study. A total T2 score, as well as individual domain scores are generated, with higher scores denoting greater social inclusion. The SInQUE takes approximately 20 minutes to complete. For the analyses reported in this paper, we used only T2 SInQUE data.

#### Manchester Short Assessment of Quality of Life (*MANSA*)

The MANSA consists of 17 items that assess overall quality of life and factors contributing to this (life domains) (Priebe et al., 1999). Items are rated from 1 (couldn’t be worse) to 7 (couldn’t be better) and a total mean score (from 1 to 7) is generated. The MANSA takes around 10 minutes to complete.

#### Brief Psychiatric Rating Scale (*BPRS*)

The BPRS is a researcher rated symptom rating tool (Overall & Gorham, 1962). Each of the 18 items are rated on a scale of 1 = not present through to 7 = severe and a total mean score from 1 to 7 is generated. The BPRS takes approximately 10 minutes to complete.

#### Discrimination and Stigma Scale (*DISC-12*)

This is a self-report scale assessing the person’s experience of stigma and discrimination comprising two subscales: experience of stigma (experienced discrimination) (21 items) and anticipation of stigma (self-stopping behaviours) (four items) (Brohan et al., 2013). Items are rated on a four point Likert scale: not at all (0); a little (1); moderately (2); a lot (3). For the purpose of this study, we assessed service users’ experiences over the past year. It takes about ten minutes to complete.

#### UCLA Loneliness Scale (*ULS-8*)

ULS-8 is a self-report measure of subjective feelings of loneliness (Hays & DiMatteo, 1987). Eight items are rated by the participant as either O (‘I often feel this way’ – score 3), S (‘I sometimes feel this way’ – score 2), R (‘I rarely feel this way’ – 1) or N (‘I never feel this way’ – score 0). The total score ranges between 0 and 32. ULS-8 has been shown to have good validity, reliability and acceptability [ref] and takes around 5 minutes to complete.

### Sample size and power

The sample size for this study was dictated by the requirements of the psychometric testing reported by Mezey et al. (2020). However, power analysis using Dunlap et al. (2004) guided our selection of independent variables for the regression modelling. It was calculated that 189 subjects would be sufficient to test for up to 11 exploratory variables with a medium (or greater) effect size (*R*^2^ = .30) with 80% power at a 5% significance level.

### Statistical analysis

Four statistical analyses were conducted to investigate the study hypotheses. The three diagnostic groups were first compared with respect to sociodemographic data, clinical variables, symptom severity, quality of life and loneliness. Continuous variables were analysed using one-way analysis of variance or Kruskal-Wallis tests (for the skewed service use variables), categorical variables by chi-squared tests. In examining how the diagnostic groups varied with respect to the SInQUE domain scores for social integration, consumption, access to services and the total SInQUE score, one-way analysis of variance was used with means and standard deviation (SD) presented for each group. The SInQUE productivity and political engagement domains were compared between diagnostic groups using Kruskal Wallis tests, and medians, (lower quartile (LQ) and upper quartile (UQ) scores are presented. These two domains had much smaller ranges and were not normally distributed, hence the need for non-parametric methods.

Multiple regression modelling was used to examine the association between current social inclusion (total SInQUE score – dependent variable) and the following variables: age; gender; ethnicity; highest education level achieved; diagnostic group [psychosis, common mental disorder, personality disorder]; number of previous admissions (as a proxy for severity of disorder); length of longest admission; experience and anticipation of stigma (DISC); and severity of psychiatric symptoms (BPRS).

The final analysis fitted two models to examine the association between current social inclusion (total SInQUE score – independent variable) and (1) quality of life (MANSA mean score) and (2) loneliness (UCL-8 total score), adjusting for socio-demographic variables, diagnostic group, number of previous admissions (as a proxy for severity of disorder); and experience and anticipation of stigma (DISC).

In all regression models, ethnicity and diagnostic group were entered as dummy variables with the largest group being used as the reference category (white and psychosis, respectively). Un-standardised regression coefficients (B) are reported for all regression models with 95% CIs. Statistical analysis was conducted using IBM SPSS for Statistics v24.

## Results

The researchers contacted all 39 eligible community mental health teams across both Trusts. Twenty-two of the teams referred a total of 238 service users to the study (nine C&I; 13 SWLSTG). Of these, six individuals did not meet the inclusion criteria, 11 could not be contacted, 28 declined to take part and one withdrew post-consent. The response rate was therefore 192/238 (80.7%).

Of the *n* = 192 participants, 106 (55%) were diagnosed as having a psychotic disorder, 49 (26%) a common mental disorder, and 37 (19%) a personality disorder. A detailed diagnostic breakdown is shown in Table 1.

**Table 1.** Detailed breakdown of diagnoses of sample, *n* = 192.

Diagnostic group	Diagnosis	*n* (%)
Psychosis	Schizophrenia	64 (33)
	Schizoaffective disorder	19 (10)
	Bipolar affective disorder	13 (7)
	Other psychotic disorders	10 (5)
Common mental disorder	Anxiety and/or depression	39 (20)
	Obsessive compulsive disorder	10 (5)
Personality disorder	Emotionally unstable personality disorder	26 (13)
	Other/non specified personality disorder	11 (6)

The mean age of participants was 42.2 years (SD 11.4, range 18 to 74 years). Over half (107; 56%) were female and two-thirds (129; 67%) were white. Descriptive statistics of participants’ socio-demographic and clinical characteristics for the whole sample and by diagnostic group can be found in Table 2.

**Table 2.** Socio-demographic characteristics, service use, severity of symptoms, quality of life and loneliness measures of sample by diagnostic group, values are *n* (%) unless otherwise stated.

		Total		Diagnostic group						F^c^, Chi^2^ or KW (*p*-value)
				Psychosis		Common mental disorder		Personality disorder		
*n*		192		106		49		37		
Age (years), mean (SD), min–max		42.4 (11.4), 18–74		43.7 (10.9), 23–74		45.1 (11.6), 18–71		35.1 (10.0), 19–52		10.5 (<.001)
Gender	Male	85	44.3%	60	56.6%	16	32.7%	9	24.3%	15.2 (<.001)
	Female	107	55.7%	46	43.4%	33	67.3%	28	75.7%	
Ethnicity	White	129	67.2%	60	56.6%	39	79.8%	30	81.1%	17.4 (.002)
	Black	37	19.3%	31	29.2%	5	10.2%	1	2.7%	
	Other	26	13.5	15	14.2%	5	10.2%	6	16.2%	
Marital status	Currently single	175	91.1%	97	91.5%	42	85.7%	36	97.3%	3.5 (0.174)
	Currently married/in a relationship	17	8.9%	9	8.5%	7	14.3%	1	2.7%	
Highest level of education reached^a^	Up to and including GCSE	104	54.2%	60	57.1%	22	45.8%	22	59.9%	2.1 (.340)
	Post GCSE	86	44.8%	45	42.9%	26	54.2%	15	40.5%	
Length of service contact (years), mean (SD), min–max		17.5 (10.6), 1–53		19.4 (10.7), 1–53		14.9 (11.2), 1–45		14.4 (8.3), 2–38		9.9 (.007)
N of previous admissions, mean (SD), min–max		4.8 (6.9) 0–50		5.6 (5.0) 1–30		1.2 (2.1) 0–12		7.2 (12.0) 0–50		55.6 (<.001)
Longest admission (months), mean (SD), min–max		10.8 (25.0), 1–204		14.4 (30.5), 1–204		4.5 (7.4), 1–36		5.0 (7.1), 1–32		7.1 (.029)
N of FTF contacts with CC/doctor in last year, mean (SD), min–max		25.6 (19.0), 3–104		22.6 (17.2), 4–104		27.2 (16.9), 3–52		31.5 (24.3), 4–104		4.0 (.139)
Psychiatric symptoms (BPRS)^b^, mean (SD), min–max		27.8 (7.1), 18–59		26.4 (7.9), 18–59		28.8 (5.1), 20–42		30.1 (6.0), 18–42		4.2 (.012),
Quality of life (MANSA), mean (SD), min–max		4.2 (1.0), 1.6–7.0		4.6 (0.9), 1.9–7.0		3.8 (1.0), 1.6–5.6		3.6 (0.9), 2.0–5.6		24.1 (<.001)
Loneliness (ULS-8), mean (SD), min–max		21.6 (5.1), 8–32		19.7 (4.9), 8–30		23.5 (4.7), 13–32		24.8 (3.7), 13–30		21.9 (<.001)

a Two missing values.

b One missing value in psychosis group.

c Continuous variables were analysed using one-way analysis of variance or Kruskal–Wallis tests, categorical variables by chi-squared tests.

The diagnostic groups differed with respect to age (*p* < .001), gender (*p* < .001), ethnicity (*p* = .002), length of contact with services (*p* < .008), number of previous admissions (*p* < .001) and longest admission (*p* = .021). Service users in the personality disorder group were younger than those in the psychosis and common mental disorder groups. There were more men in the psychosis group and more women in the other two groups. Around 80% of individuals in the common mental disorder and personality disorder groups were white, as compared to fewer than 60% of the psychosis group. The mean duration of contact with services was nearly twenty years for individuals in the psychosis group, approximately 5 years longer than the other two groups. Individuals in the common mental disorder group had the fewest previous admissions and the personality disorder group had the most. The mean length of longest admission was substantially greater in the psychosis group than in the other two groups.

Individuals in the personality disorder group scored highest for symptom severity (BPRS) and those in the psychotic group scored lowest (*p* = .012)

### Do diagnostic groups differ with respect to current social inclusion?

The three diagnostic groups did not differ significantly with respect to their SInQUE scores on four out of the five domains or the total score. There was, however, a significant difference in the Productivity domain score (i.e. paid employment, voluntary work etc), with the personality disorder group scoring highest (*p* = 0.002) that is being most socially included on this domain (Table 3).

**Table 3.** Comparison of current (T2) SInQUE scores between diagnostic groups, *n* = 192.

	Total	Psychosis	Common mental disorder	Personality disorder	*F*^a^ or KW (*p*-value)
Social integration	19.3 (5.7)	19.7 (6.0)	18.9 (5.2)	18.8 (5.4)	0.5 (.628)
Consumption	16.1 (3.3)	16.1 (3.6)	16.2 (3.2)	16.2 (2.6)	0.0 (.982)
Productivity	0.0 (0.0, 1.0)	0.0 (0.0, 1.0)	0.0 (0.0, 0.0)	1.0 (0.0, 1.0)	12.4 (.002)
Access to services	2.9 (0.9)	2.8 (0.9)	3.1 (0.8)	3.0 (0.8)	1.5 (.218)
Political engagement	1.0 (1.0, 1.0)	1.0 (1.0, 1.0)	1.0 (1.0, 1.0)	1.0 (0.0, 1.0)	0.5 (.777)
Total SInQUE	39.8 (8.6)	39.9 (9.1)	39.5 (8.1)	39.9 (7.8)	0.0 (.967)

a Kruskall–Wallis KW statistic for productivity and political engagement domains, one way analysis of variance *F* statistic for other domains and total score.

### Factors associated with social inclusion

Lower social inclusion (as assessed by the total SInQUE score) was associated with older age (*p* = .011), lack of education beyond GCSE level (*p* < .001), more previous admissions (*p* = .007), greater experience of stigma (*p* = .009) and anticipation of stigma (*p* = .041) and greater severity of psychiatric symptoms (0.001) (Table 4). Gender, ethnicity and diagnostic group were not associated with the degree of social inclusion.

**Table 4.** Factors associated with current social inclusion (T2 SInQUE total score).

		*n* = 188 (*R*^2^ = 0.293)		
		*B*	95% CI	*p*-Value
Age (years)		−0.1	−0.2, 0.0	.011
Gender	Female	0	–	–
	Male	−0.3	−2.6, 2.1	.811
Ethnic group	White	0	–	–
	Black	−2.5	−5.5, 0.4	.092
	Other	−2.3	−5.6, 0.9	.162
Education	Past GCSE	0	–	–
	Up to GCSE	−4.3	−6.6, −2.0	<.001
Diagnosis	Psychotic disorder	0	–	–
	Common mental disorder	−0.8	−3.7, 2.0	.565
	Personality disorder	1.7	−1.6, 5.1	.313
Number of previous admissions		−0.2	−0.4, −0.1	.007
Experience of stigma (total)		−0.4	−0.7, −0.1	.009
Anticipated stigma (total)		−1.0	−1.9, 0.0	.041
Psychiatric symptoms (BPRS)		−0.3	−0.4, −0.1	.001

### Association between social inclusion, quality of life and loneliness

Greater social inclusion was associated with better quality of life (*p* < .001) (Table 5) and less loneliness (*p* < .001). Compared with the participants in the psychosis group, those in the common mental disorder and personality disorder groups had poorer quality of life (both *p* < .001) and were lonelier (both *p* = .001), even after adjustment for confounding variables. Individuals with common mental disorders scored between the other two groups on both measures.

**Table 5.** Association between current social inclusion (T2 SInQUE total score) and quality of life (MANSA) and loneliness (UCL-8).

		Quality of life (*R*^2^ = 0.587)			Loneliness (*R*^2^ = .533)		
		*B*	95% CI	*p*-Value	*B*	95% CI	*p*-Value
Age (years)		0.0	0.0, 0.0	.108	0.0	0.0, 0.1	.888
Gender	Female	0	–	–	0	–	–
	Male	0.1	−0.1, 0.3	.239	−0.9	−2.1, 0.2	.109
Ethnic group	White	0	–	–	0	–	–
	Black	0.2	−0.1, 0.5	.130	−1.3	−2.8, 0.1	.067
	Other	−0.1	−0.4, 0.2	.397	0.8	−0.8, 2.4	.342
Education	Past GCSE	0	–	–	0	–	–
	Up to GCSE	0.1	−0.1, 0.2	.553	0.6	−0.6, 1.7	.316
Diagnosis	Psychotic disorder	0	–	–	0	–	–
	Common mental disorder	−0.6	−0.9, −0.4	<.001	2.3	0.9, 3.7	.001
	Personality disorder	−0.7	−2.5, 4.3	<.001	2.7	1.1, 4.3	.001
Number of previous admissions		0.0	0.0, 0.0	.094	−0.1	−0.2, 0.0	.055
Experience of stigma (total)		−0.1	−0.1, 0.0	<.001	0.4	0.2, 0.6	<.001
Anticipated stigma (total)		−0.1	−0.2, 0.0	.078	0.7	0.3, 1.2	.001
Total SInQUE T2		0.1	0.0, 0.1	<.001	−0.2	−0.2, −0.1	<.001

## Discussion

We hypothesised that levels of social inclusion would vary according to the type of mental disorder. However, there was, overall, very little difference in SInQUE scores across the three diagnostic categories. All the participants in this study had severe mental disorder, as evidenced by the fact that they were under the care of secondary or tertiary mental health services and had all had all had at least one previous hospital admission or been referred to a home treatment team. This finding would suggest that the severity of mental disorder has a greater impact on an individual’s social inclusion than the specific diagnosis. However, we cannot say from our results whether similar results would be found in individuals with less severe mental disorders, including those under the care of their GP.

Lower social inclusion was associated with both experienced and anticipated stigma. This suggests that fear of encountering negative or rejecting responses to disclosures of mental health problems may curtail people’s willingness to participate in social, recreational and work opportunities, resulting in social withdrawal and exclusion.

Lower social inclusion was also associated with a greater number of previous admissions and more severe current psychiatric symptoms. Repeated psychiatric admissions, particularly from a young age, would be expected to have a disruptive effect on developing social relationships, work and educational attainment, as well as being indicative of a more severe or treatment resistant illness trajectory. The association between more severe and longer term illness and lower social inclusion makes intuitive sense since those with more severe symptoms (such as hallucinations and delusions) and/or negative symptoms, which impact on behaviour, motivation, interpersonal skills and day to day function, are likely to encounter more stigma and discrimination from others, making it more difficult to engage with social, educational or vocational activities (Broussard et al., 2012; Stuart & Arboleda-Florez, 2001).

Having no qualifications beyond GCSEs was also significantly associated with lower social inclusion. More long term and severe illness is likely to be associated with disrupted education, causing a negative impact on various aspects of later social inclusion, such as employability and consumption.

As hypothesised, service users who reported greater current social inclusion also reported better quality of life and less loneliness. This would suggest that increased social inclusion, as well as being valued by service users, should also be a desirable treatment goal for practitioners. People with personality disorder reported feeling more lonely than individuals in the other two groups, despite scoring higher on the Productivity domain of social inclusion. Interpersonal problems and problems with social relationships are a common feature of personality disorder, so this finding is perhaps not surprising. However, this finding also appears to reinforce the notion that that loneliness may be driven by the perceived quality of relationships and not just the quantity of social contact (Perlman & Peplau, 1981).

There were a number of socio-demographic and clinical differences between the three diagnostic groups. Individuals with psychosis had more indicators of social deprivation and disadvantage than those in the other two groups. For example, one third had no qualifications compared to 14.3% of those with a common mental disorder and 16.2% of those with a personality disorder. They were also less likely to be in paid employment (8.5% psychosis vs. 12.2% common mental disorder vs. 21.6% personality disorder) and they were significantly more likely to be from black and minority ethnic groups (29.2% vs. 10.2% vs. 2.7% respectively). All of these factors are likely to impact on social inclusion and were therefore adjusted for in our final analysis.

### Strengths and limitations

Although all participants in the study had severe mental health problems, the most mentally unwell individuals may have been excluded from the study, due to concerns by their care co-ordinators about their ability to participate and/or their lack of capacity to give informed consent for participation. It may be that these individuals are at the greatest risk of social exclusion, given the trend for more severe symptoms being associated with low levels of social inclusion.

However this is one of the few studies that have included different diagnostic groups.

Our model explained just under 30% of the variance in participants’ social inclusion, quality of life and loneliness. Clearly, other unmeasured factors, such as personality type, social circumstances and neighbourhood characteristics or the presence of specific symptoms, not reflected in the overall BPRS symptom severity score, may also have contributed to overall SInQUE score.

The BPRS was used to assess severity of symptoms across the three diagnostic groups, although its validity and reliability has mainly been established for individuals with psychotic illnesses. We are therefore aware that caution should be applied when interpreting the relationship between the association of severity of symptoms and social inclusion in the common mental disorders and personality disorder groups.

## Implications

Social inclusion is worth pursuing both as an end in itself but also because of its association with greater quality of life and less loneliness in individuals with severe mental illness.

This study does not support the need for bespoke social inclusion interventions for different diagnostic groups. However, there is a clear need for the holistic, bio-psychosocial approach of generic mental health services to include assessing and addressing social inclusion as a routine aspect of mental health care across all diagnoses. Our findings suggest that specific characteristics, rather than diagnostic groupings, should be taken account of as part of this assessment, such as length of history, educational achievement, recurrent admissions and current symptoms. Whilst societal stigma and discrimination may exacerbate social exclusion, it is nevertheless incumbent upon mental health practitioners to identify and address factors that can facilitate greater social inclusion, including more effective management of symptoms.

## Research implications

Future studies could aim to establishing population norms for social inclusion and to explore differences in social inclusion in more specific clinical groups and service settings.

Longitudinal studies are required to investigate further the direction of effect of characteristics associated with social inclusion in order to inform which predictive factors are most amenable to intervention.

There is a need to develop and evaluate specific interventions to enhance social inclusion for people with severe mental health problems (Webber & Fendt-Newlin, 2017). Social inclusion is also a relevant outcome for studies of interventions with a primary aim of reducing symptoms, as the association found in our study suggests this is a promising way to reduce social exclusion.

Further studies are required to understand whether the SInQUE, as a new measure of social inclusion, can be implemented in routine mental health practice, to assess its use as a care planning tool and to assess whether it is effective in terms of impact on service user outcomes related to social inclusion.
